# Supplementary material for: Investigation of commonly used aortic aneurysm growth rate metrics: Comparing their suitability for clinical and research applications
Source: PLoS One. 2023 Aug 11;18(8):e0289078. doi: 10.1371/journal.pone.0289078 (PMC10420361; doi:10.1371/journal.pone.0289078)
Supplement: S1 Appendix — (DOCX) [file pone.0289078.s001.docx]

**A. STAN code for linear mixed model**

varying_intercept_slope = """

data {

  int<lower=0> N;

  int<lower=0> J;

  vector[N] y;

  vector[N] x;

  int mrn[N];

}

parameters {

  real<lower=0> sigma;

  real<lower=0> sigma_a;

  real<lower=0> sigma_b;

  vector[J] a;

  vector[J] b;

  real mu_a;

  real mu_b;

}

model {

  mu_a ~ normal(0, 100);

  mu_b ~ normal(0, 100);

  a ~ normal(mu_a, sigma_a);

  b ~ normal(mu_b, sigma_b);

  y ~ normal(a[mrn] + b[mrn].*x, sigma);

} """

**B. OpenBUGS code for exponential mixed model**

model

{

for( i in 1 : N ) {

y[i] ~ dnorm(mu[i],tau.c)

mu[i] <- exp( beta[patid[i]] * (x[i] + alpha[patid[i]]) ) + 2

}

for(R in 1:Npats){

alpha[R] ~ dnorm(alpha.c,alpha.tau)

beta[R] ~ dnorm(beta.c,beta.tau)

}

tau.c ~ dgamma(0.001,0.001)

alpha.c ~ dnorm(0.0,100)

alpha.tau ~ dgamma(0.001,0.001)

beta.c ~ dnorm(0.0,100)

beta.tau ~ dgamma(0.001,0.001)

alpha0 <- alpha.c - beta.c

}
